# Lifecourse Neighborhood Disadvantage and Global Cognition in a Community-Based Study of Black Adults

**DOI:** 10.1093/geroni/igaf122.742

**Published:** 2025-12-31

**Authors:** Andrea Rosso, Greta Jianjia Cheng, Gerald Hunter, Tanisha Hill-Jarrett, Bonnie Dastidar, Wendy Troxel, Tamara Dubowitz

**Affiliations:** University of Pittsburgh, Pittsburgh, Pennsylvania, United States; University of Pittsburgh, Pittsburgh, Pennsylvania, United States; RAND, Pittsburgh, Pennsylvania, United States; University of California Memory and Aging Center, San Francisco, California, United States; RAND, Santa Monica, California, United States; RAND, Pittsburgh, Pennsylvania, United States; University of Pittsburgh School of Public Health, Pittsburgh, Pennsylvania, United States

## Abstract

Evidence suggests that neighborhood characteristics affect cognitive health in later life. Nearly all existing studies have assessed neighborhood context at study enrollment and lack a lifecourse perspective. For cognitive aging, early life exposures may play a critical role. We obtained lifetime residential histories from participants in the Think PHRESH study, a cohort of participants (n = 423, mean age=63, 75% female, 95% Black) from two historically disinvested, predominantly Black neighborhoods in Pittsburgh, PA. Participants were randomly sampled from a complete list of addresses in each neighborhood. Addresses were linked to historical census data to quantify neighborhood deprivation using 14 census measures across five lifecourse periods (childhood, adolescence, young adulthood, middle age, late life). Linear models estimated associations of deprivation at each lifecourse period with current Modified Mini Mental State Examination (3MS) scores, adjusted for demographic characteristics. Mean 3MS score was 89 (SD = 8). Greater deprivation in childhood and adolescence was not related to 3MS scores. Greater neighborhood deprivation during young adulthood (beta=-1.59 (95% CI: -2.72, -0.46)) and midlife (beta=-1.63 (95% CI: -3.00, -0.28)) was related to lower 3MS scores. Greater deprivation in late life was marginally related to lower 3MS scores (beta=-1.79 (95% CI: -3.65, 0.08)). These results highlight young adulthood and midlife as potential critical windows of exposure to neighborhood deprivation for later life global cognitive functioning and may suggest specific pathways by which these exposures influence dementia risk.

